# Lipid accumulation product index is inversely U-shaped associated with abdominal aortic calcification based on NHANES 2013–2014

**DOI:** 10.3389/fcvm.2025.1524847

**Published:** 2025-06-09

**Authors:** Jiangbei Deng, Xiao Qin

**Affiliations:** ^1^ Department of Vascular Surgery, The First Affiliated Hospital of Guangxi Medical University, Nanning, China; ^2^ The Affiliated Changsha Central Hospital, Department of Peripheral Vascular Intervention, Hengyang Medical School, University of South China, Hengyang, China

**Keywords:** lipid accumulation product, abdominal aortic calcification, visceral obesity index, NHANES, cross-sectional study

## Abstract

**Background:**

In this study, we explored the correlation between lipid accumulation product (LAP) and abdominal aortic calcification (AAC).

**Methods:**

Data collected from 2013–2014 were obtained from the National Health and Nutrition Examination Survey (*N*HANES) database. We utilized weighted univariate and multivariate regression analyses to assess the correlation between ln-LAP (LAP was transformed using a natural logarithm) and AAC. Further, subgroup analyses, smoothed curve fitting, and sensitivity analysis were implemented.

**Results:**

The study included 2,965 participants, with a mean ln-LAP index of 3.95 ± 0.83. Following adjustment for all covariates, multiple regression analyses indicated that ln-LAP, when modeled as a quadratic categorical variable, was significantly positively associated with AAC in Q3 (OR = 1.91; 95% CI: 1.20, 3.04, *P* < 0.001) compared to the Q1, and similarly, with severe abdominal aortic calcification (SAAC) in Q4 (OR = 2.17; 95% CI: 1.08, 4.35, *P* < 0.05). Conversely, Q2, Q3, and Q4 did not exhibit significant positive correlations with AAC scores (*P* > 0.05). Smoothed curve fitting revealed a nonlinear relationship between ln-LAP and AAC, characterized by an inverse U-shaped curve. Threshold effect analysis identified an inflection point at 4.21. Before this point, a marked positive correlation existed between ln-LAP and AAC (OR=1.74); beyond this point, a pronounced negative correlation was observed (OR=0.60). Subgroup analyses revealed no significant interactions regarding the correlation across age, sex, hypertension, and diabetes groups (*P* interaction >0.05).

**Conclusions:**

This research reveals a significant inverse U-shaped correlation between LAP and the prevalence of AAC, implying that LAP could serve as a potential biomarker for evaluating AAC risk.

## 1 Introduction

Abdominal aortic calcification (AAC) consists of calcified lesions forming within the abdominal aortic walls (1). These lesions are predominantly observed in the elderly, with their prevalence escalating with age (2), and serve as critical predictors of cardiovascular disease. Research has demonstrated that AAC is strongly associated with all-cause mortality, cardiovascular events, and cardiovascular mortality (3, 4). Therefore, the detection and management of AAC are crucial for reducing the risk of cardiovascular disease. However, the detection of AAC in large-scale populations is limited by the complex nature of these methods and their primary reliance on imaging. Consequently, identifying a simple, user-friendly biomarker for predicting AAC holds considerable research value.

LAP, emerging as a novel obesity assessment index, outperforms traditional obesity indicators in predicting cardiovascular risk factors and metabolic syndrome (5, 6). Should LAP be associated with AAC, it may serve as a potential early predictive biomarker for this condition. This association could facilitate the identification of high-risk individuals and provide a foundation for clinical interventions aimed at reducing the incidence of cardiovascular events. Until now, no research has comprehensively analyzed the relationship between LAP and AAC. In this study, we employed data from the 2013–2014 National Health and Nutrition Examination Survey (NHANES) to elucidate the relationship between LAP and AAC and to assess its predictive validity for AAC. The findings of this research are expected to provide novel insights into the early detection of AAC and further validate the potential clinical applications of LAP in related cardiovascular pathologies.

## 2 Materials and methods

### 2.1 Data source and participants

**Figure 1:**
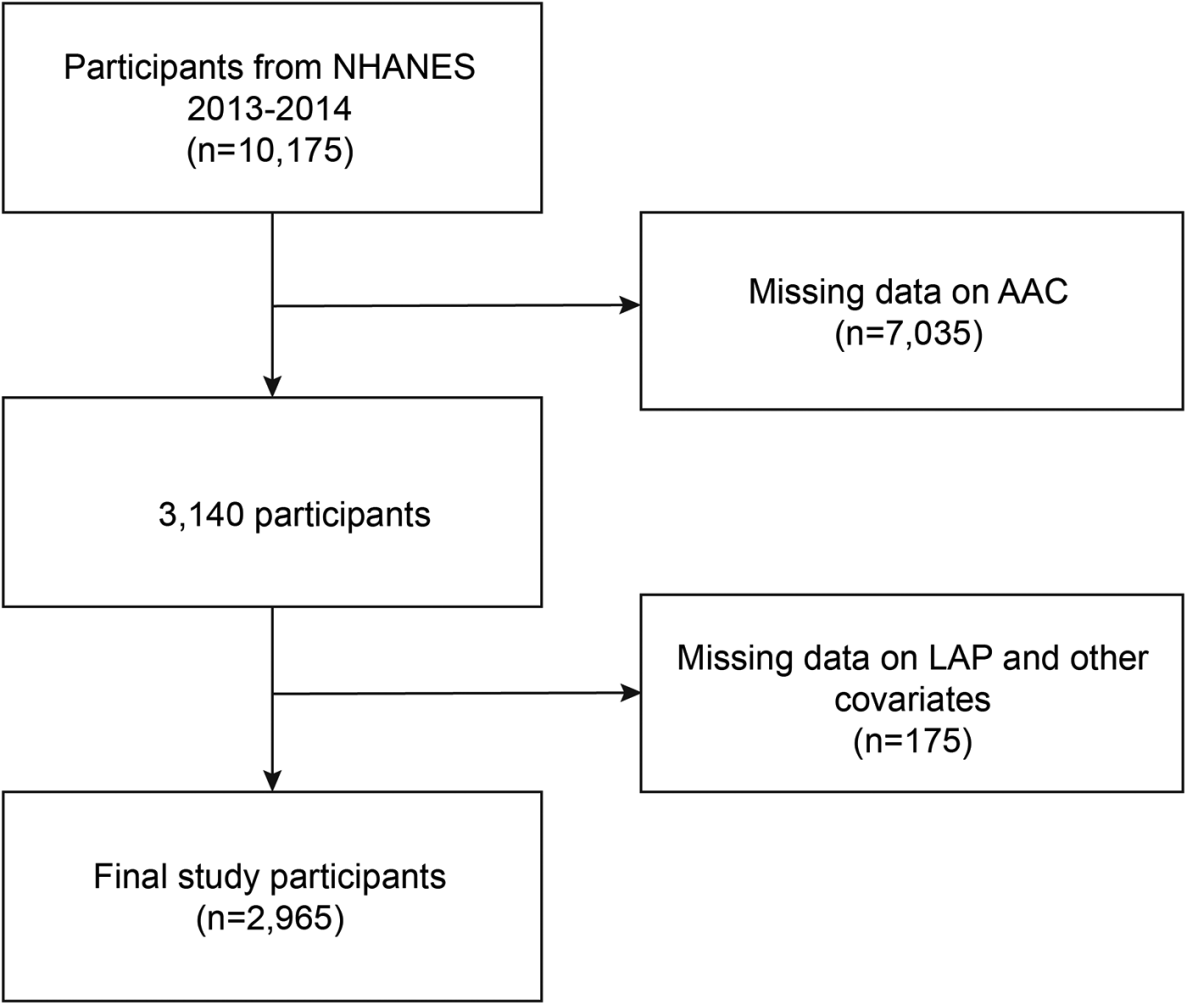
Flowchart of participant selection.

The NHANES is a comprehensive assessment conducted in the United States, aimed at gathering data from a representative cohort of adults and children encompassing demographic, health behavior, and nutritional information. Data from NHANES are accessible via https://www.cdc.gov/nchs/nhanes, and the initiative has secured ethical review approval from the National Center for Health Statistics (NCHS). This investigation utilized data from the 2013–2014 NHANES, with an initial sample comprising 10,175 participants. The final cohort included 2,965 participants, following the exclusion of 7,035 due to missing AAC data and an additional 175 for lacking LAP and other covariate data. The process of sample selection is depicted in Figure 1.

### 2.2 Definition of AAC

AAC assessment was conducted via lateral dual-energy x-ray absorptiometry (DXA) scanning of vertebrae L1-L4, following the Kauppila scoring system. The study revealed that lateral spinal DXA imaging exhibits high specificity and sensitivity in AAC diagnosis, while also minimizing radiation exposure to patients. The Kauppila scores varied from 0–24; scores exceeding 0 indicate AAC, while those over 6 suggest SAAC presence (7, 8).

### 2.3 Definition of LAP Index

In the study, LAP was utilized as an exposure factor. The LAP was calculated using the formula [Waist Circumference (WC) (cm)—65] × [Triglycerides (TG) (mmol/L)] for males and [WC (cm)—58] × [TG (mmol/L)] for females. Owing to its non-normal distribution, the LAP index underwent a natural logarithm transformation (ln-transformed LAP) (9).

### 2.4 Covariates

This study incorporated a range of potential covariates, including age, gender (male, female), race (Mexican American, other Hispanic, non-Hispanic white, non-Hispanic black, other), educational attainment (less than high school diploma, high school graduate or GED, beyond high school), family poverty level (FPL) (<1.3, 1.3–3.5, ≥3.5), marital status (married or cohabitating; widowed, divorced, or separated; never married), body mass index (BMI) (<25, 25–30, ≥30 kg/m^2^), hypertension, diabetes, smoking status, alcohol consumption, hyperuricemia (male ≥420 μmol/L, female ≥360 μmol/L), and sedentary behavior (SB). Demographic data, including age, sex, race, education level, annual household income to poverty ratio, and marital status, were collected via household interviews. Weight, height, and WC measurements were conducted during the mobile medical examinations. Hypertension was defined as either a physician's diagnosis or the use of antihypertensive medication. Diabetes was defined as either a physician's diagnosis or the administration of insulin or antidiabetic medication. Alcohol consumption was quantified as the intake of five or more alcoholic beverages per day. Smoking status was characterized by the consumption of at least 100 cigarettes over a lifetime. Hypercholesterolemia was defined based on either a physician's notification of elevated cholesterol levels or serum cholesterol exceeding 240 mg/dl. SB was delineated as time spent engaged in predominantly seated activities, including reading, playing cards, watching television, using a computer, or traveling by car.

### 2.5 Statistical analyses

Statistical analyses in this study were performed using the R software package (R Foundation: http://www.r-project.org; version 3.4.3) and Empower (R) (https://www.empowerstats.com, X&Y Solutions, Inc., Boston, Massachusetts Massachusetts)). Considering the complex multistage sampling design of NHANES, this study utilized mobile examination center examination weights (WTMEC2YR) for analysis. In this analysis, categorical variables were expressed as weighted percentages, while continuous variables were depicted using weighted means and standard deviations. Group differences were assessed using chi-square tests for categorical variables and one-way analysis of variance (ANOVA) for continuous variables. All statistical tests conducted were two-sided, and a *p*-value of less than 0.05 was deemed statistically significant. Furthermore, a weighted multivariable logistic regression model was employed to explore the correlation between ln-LAP and AAC, with ln-LAP stratified into quartiles. Three analytical models were applied: Model 1 without covariate adjustments; Model 2 adjusted for gender, age, and race; and Model 3 comprehensively adjusted for age, gender, race, educational level, FPL, marital status, BMI, hypertension, diabetes, smoking, alcohol consumption, hyperuricemia, and SB. Smooth curves were constructed to visually illustrate the relationship between ln-LAP and AAC; these graphs feature a solid red line representing the estimated values and blue dashed areas indicating the 95% confidence intervals. Subgroup analyses were performed to ascertain whether the relationship between ln-LAP and AAC varied across gender, age, hypertension, and diabetes categories. Sensitivity analysis was performed by excluding participants with extreme values of ln-LAP (<2 or ≥6) to test the robustness of the results.

## 3 Results

### 3.1 Participant characteristics

This study encompassed 2,965 participants from the NHANES 2013–2014 dataset, featuring a mean weighted age of 57.43 ± 11.52 years, with 48.59% males and 51.41% females. The mean ln-LAP was 3.98 ± 0.83, stratified into quartiles: 0.28–3.42, 3.42–3.97, 3.97–4.54, and 4.54–8.24. Significant differences were observed between ln-LAP and AAC scores, as well as between AAC and SAAC across ln-LAP quartiles. With increasing ln-LAP levels, these indicators correspondingly increased. Significant differences were noted among quartiles in terms of gender, race, education level, FPL, BMI, hypertension, diabetes, smoking, alcohol consumption, hyperuricemia, WC, as well as systolic blood pressure, high-density lipoprotein (HDL), low-density lipoprotein (LDL), TG, aspartate aminotransferase (AST), and alanine aminotransferase (ALT) levels. However, no significant differences were found among quartiles regarding marital status, sleep duration, and SB (Table 1).

**Table 1:** Characteristics of study participants.

Characteristics^a^	ln-LAP				*P* value
	Q1	Q2	Q3	Q4	
	(0.28–3.42)	(3.42–3.97)	(3.97–4.54)	(4.54–8.24)	
Age (years) (%)	55.71 ± 11.55	58.24 ± 11.74	57.90 ± 11.70	57.84 ± 10.90	<0.0001
Gender (%)					<0.0001
Male	42.69	47.55	48.2	55.54	
Female	57.31	52.45	51.8	44.46	
Race (%)					<0.0001
Mexican American	4.04	6.07	8.83	8.77	
Other Hispanic	4.16	4.83	4.87	4.88	
Non-Hispanic white	70	68.84	71.54	74.85	
Non-Hispanic black	13.04	11.93	8.66	5.89	
Other race	8.76	8.33	6.11	5.62	
Education level (%)					<0.001
Less than high school	13.11	14.44	16.32	16.92	
High school	18.51	20.62	21.94	25.97	
More than high school	68.37	64.94	61.74	57.11	
BMI (kg/m^2^) (%)					<0.0001
<25	64.6	26.2	13.91	3.6	
25–30	28.98	50.87	40.28	29.7	
≥30	6.43	22.93	45.81	66.7	
Marital status					0.83
Married or cohabitating	69.17	69.65	68.52	68.89	
Widowed, divorced, or separated	22.98	24.2	24.98	24.78	
Never married	7.86	6.15	6.5	6.33	
FPL (%)					<0.0001
<1	18.62	16.56	19.13	21.78	
1–3	26.41	33.28	34.97	38.8	
≥3	54.97	50.16	45.91	39.43	
Smoking (%)					<0.0001
No	61.66	55.48	52.72	47.88	
Yes	38.34	44.52	47.28	52.12	
Alcohol consumption (%)					<0.0001
No	87.41	83.95	82.49	77.3	
Yes	12.59	16.05	17.51	22.7	
Diabetes (%)					<0.0001
No	94.81	89.93	86.02	75.27	
Yes	5.19	10.07	13.98	24.73	
Hypertension (%)					<0.001
No	26.85	19.49	14.56	13.45	
Yes	73.15	80.51	85.44	86.55	
Hyperuricemia (%)					<0.0001
No	92.28	86.12	82.99	70.99	
Yes	7.72	13.88	17.01	29.01	
Sleep time (h) (%)					0.10
<7	30.86	36.81	31.3	36.1	
7–9	60.81	56.29	60.55	55.54	
≥9	8.33	6.9	8.16	8.36	
AAC					<0.0001
No	78.36	68.74	69.41	68.44	
Yes	21.64	31.26	30.59	31.56	
SAAC					<0.05
No	94.98	90.72	91.7	91.32	
Yes	5.02	9.28	8.3	8.68	
AAC score	1.07 ± 2.86	1.61 ± 3.33	1.56 ± 3.38	1.63 ± 3.48	<0.01
SB (min)	435.10 ± 589.51	449.69 ± 544.51	444.65 ± 443.08	485.47 ± 671.65	0.34
SBP (mmHg)	121.26 ± 17.90	126.47 ± 18.70	125.14 ± 16.74	128.21 ± 16.35	<0.0001
HDL (mmol/L)	1.77 ± 0.50	1.50 ± 0.37	1.31 ± 0.30	1.10 ± 0.26	<0.0001
LDL (mmol/L)	2.78 ± 0.83	3.02 ± 0.93	3.17 ± 1.00	3.07 ± 0.98	<0.0001
TG (mmol/L)	0.80 ± 0.29	1.20 ± 0.35	1.81 ± 0.56	3.34 ± 2.64	<0.0001
AST (U/L)	25.02 ± 12.35	24.24 ± 13.53	24.84 ± 12.04	27.62 ± 20.20	<0.0001
ALT (U/L)	21.74 ± 15.44	23.20 ± 20.46	25.20 ± 14.99	28.76 ± 17.33	<0.0001

^a^ Data are presented as (%) or mean ± standard deviation.

Q1, Quartile 1; Q2, Quartile 2; Q3, Quartile 3; Q4, Quartile 4; ln-LAP, ln-transformed lipid accumulation product; AAC, abdominal aortic calcification; SAAC, severe abdominal aortic calcification; FPL, family poverty level; BMI, body mass index; SB, sedentary behavior; SBP, systolic blood pressure; HDL, high-density lipoprotein; LDL, low-density lipoprotein; TG, triglyceride; AST, aspartate aminotransferase; ALT, alanine aminotransferase.

### 3.2 Associations between ln-LAP and AAC score, AAC, SAAC

According to Table 2, in the unadjusted model, dividing ln-LAP into four quartiles revealed significant positive correlations with AAC scores in Q2 (*β* = 0.54; 95% CI: 0.20, 0.88; *P* < 0.01), Q3 (*β* = 0.49; 95% CI: 0.15, 0.82; *P* < 0.01), and Q4 (*β* = 0.56; 95% CI: 0.23, 0.89; *P* = 0.001). Furthermore, significant positive correlations with AAC in Q2 (OR = 1.41; 95% CI: 1.12, 1.77; *P* < 0.01), Q3 (OR = 1.52; 95% CI: 1.21, 1.90; *P* < 0.001), and Q4 (OR = 1.36; 95% CI: 1.08, 1.71; *P* < 0.01) were identified, as well as with SAAC for Q3 (OR = 1.46; 95% CI: 1.00, 2.12; *P* < 0.05) and Q4 (OR = 1.50; 95% CI: 1.03, 2.17; *P* < 0.05). With further adjustments for age, gender, race, educational level, FPL, marital status, BMI, hypertension, diabetes, smoking, alcohol consumption, hyperuricemia, and SB, dividing ln-LAP into four quartiles showed that, compared to Q1, significant positive correlations with AAC in Q3 (OR = 1.91; 95% CI: 1.20, 3.04; *P* < 0.01) and with SAAC in Q4 (OR = 2.17; 95% CI: 1.08, 4.35; *P* < 0.05) were observed. No significant positive correlations with AAC scores were observed in Q2, Q3, and Q4 (*P* > 0.05).

**Table 2:** Association between ln-LAP and AAC score, AAC and SAAC.

Variable	Model 1	*P*-value	Model 2	*P*-value	Model 3	*P*-value
	β/OR (95% CI)		β/OR (95% CI)		β/OR (95% CI)	
AAC score						
ln-transformed LAP (categories)						
Q1	0		0		0	
Q2	0.54 (0.20, 0.88)	<0.01	0.26 (−0.06, 0.57)	0.11	0.33 (−0.43, 1.09)	0.40
Q3	0.49 (0.15, 0.82)	<0.01	0.24 (−0.07, 0.55)	0.13	0.42 (−0.38, 1.23)	0.30
Q4	0.56 (0.23, 0.89)	<0.01	0.32 (0.01, 0.63)	0.04	0.44 (−0.37, 1.26)	0.29
AAC						
ln-transformed LAP (categories)						
Q1	1		1		1	
Q2	1.41 (1.12, 1.77)	<0.01	1.28 (1.00, 1.63)	<0.05	1.18 (0.76, 1.84)	0.47
Q3	1.52 (1.21, 1.90)	<0.001	1.39 (1.09, 1.78)	<0.01	1.91 (1.20, 3.04)	<0.01
Q4	1.36 (1.08, 1.71)	<0.01	1.25 (0.98, 1.60)	0.07	1.38 (0.84, 2.28)	0.20
SAAC						
ln-transformed LAP (categories)						
Q1	1		1		1	
Q2	1.39 (0.95, 2.03)	0.09	1.19 (0.79, 1.79)	0.41	1.38 (0.74, 2.56)	0.31
Q3	1.46 (1.00, 2.12)	<0.05	1.26 (0.84, 1.90)	0.26	1.61 (0.84, 3.05)	0.15
Q4	1.50 (1.03, 2.17)	<0.05	1.43 (0.96, 2.15)	0.08	2.17 (1.08, 4.35)	<0.05

Model 1 was adjusted for none. Model 2 was adjusted for age, gender, and race. Model 3 was adjusted for age, gender, race, education level, FPL, marital status, BMI, hypertension, diabetes, smoking, alcohol consumption, hyperuricemia, and SB. Q1, Quartile 1; Q2, Quartile 2; Q3, Quartile 3; Q4, Quartile 4; ln-LAP, ln-transformed lipid accumulation product; AAC, abdominal aortic calcification; SAAC, severe abdominal aortic calcification; FPL, family poverty level; BMI, body mass index; SB, sedentary behavior.

### 3.3 Stratified analyses

As illustrated in Table 3, Our study examined the association between ln-LAP and both AAC and SAAC across various subgroups defined by age, gender, hypertension, and diabetes. The analyses were adjusted for multiple covariates including age, gender, race, education level, federal poverty level (FPL), marital status, BMI, hypertension, diabetes, smoking status, alcohol consumption, hyperuricemia, and systolic blood pressure (SB). Subgroup analyses revealed that the correlation between ln-LAP and both AAC and SAAC was consistent across age, gender, hypertension, and diabetes subgroups, exhibiting no significant interaction (*P* > 0.05 for interaction).

**Table 3:** Subgroup analysis of ln-LAP and AAC score, AAC and SAAC.

ln-LAP	Q1	Q2	*P*-value	Q3	*P*-value	Q4	*P*-value	*P* for interaction
AAC								
Gender								
Male	Reference	1.15 (0.62, 2.12)	0.66	2.29 (1.24, 4.21)	<0.01	1.06 (0.55, 2.01)	0.87	0.13
Female	Reference	1.18 (0.63, 2.22)	0.61	1.47 (0.76, 2.84)	0.25	1.65 (0.84, 3.23)	0.14	
Age								
<60	Reference	1.27 (0.56, 2.84)	0.57	2.05 (0.91, 4.62)	0.08	1.12 (0.49, 2.56)	0.79	0.89
>=60	Reference	1.19 (0.72, 1.97)	0.49	1.73 (1.03, 2.91)	<0.05	1.28 (0.74, 2.22)	0.37	
Hypertension								
No	Reference	1.02 (0.40, 2.64)	0.96	1.94 (0.71, 5.30)	0.19	1.97 (0.74, 5.21)	0.17	0.68
Yes	Reference	1.20 (0.73, 1.98)	0.46	1.83 (1.10, 3.05)	<0.05	1.26 (0.73, 2.17)	0.41	
Diabetes								
No	Reference	1.21 (0.74, 1.97)	0.44	1.94 (1.16, 3.22)	<0.05	1.39 (0.80, 2.40)	0.24	0.97
Yes	Reference	0.95 (0.32, 2.79)	0.92	1.46 (0.50, 4.23)	0.49	1.12 (0.40, 3.15)	0.83	
SAAC								
Gender								
Male	Reference	0.91 (0.39, 2.09)	0.82	1.14 (0.49, 2.64)	0.76	1.11 (0.45, 2.77)	0.82	0.50
Female	Reference	1.82 (0.72, 4.60)	0.21	1.91 (0.74, 4.91)	0.18	2.82 (1.08, 7.36)	0.03	
Age								
<60	Reference	1.97 (0.36, 10.93)	0.44	2.16 (0.36, 12.84)	0.40	1.16 (0.19, 7.04)	0.87	0.72
>=60	Reference	1.12 (0.60, 2.11)	0.72	1.21 (0.63, 2.30)	0.57	1.34 (0.67, 2.67)	0.41	
Hypertension								
No	Reference	1.03 (0.25, 4.21)	0.97	1.35 (0.30, 6.04)	0.69	1.44 (0.33, 6.25)	0.63	0.99
Yes	Reference	1.29 (0.65, 2.55)	0.47	1.45 (0.72, 2.92)	0.29	1.83 (0.86, 3.86)	0.11	
Diabetes								
No	Reference	1.49 (0.75, 2.95)	0.25	1.49 (0.73, 3.05)	0.27	1.51 (0.68, 3.34)	0.31	0.35
Yes	Reference	0.64 (0.15, 2.68)	0.54	1.12 (0.28, 4.52)	0.88	1.84 (0.47, 7.12)	0.38	

Adjusted for gender, age, gender, race, education level, FPL, marital status, BMI, hypertension, diabetes, smoking, alcohol consumption, hyperuricemia, SB. Q1, Quartile 1; Q2, Quartile 2; Q3, Quartile 3; Q4, Quartile 4; ln-LAP, ln-transformed lipid accumulation product; AAC, abdominal aortic calcification; SAAC, severe abdominal aortic calcification; FPL, family poverty level; BMI, body mass index; SB, sedentary behavior.

### 3.4 Dose-relationship between ln-LAP and AAC

**Figure 2:**
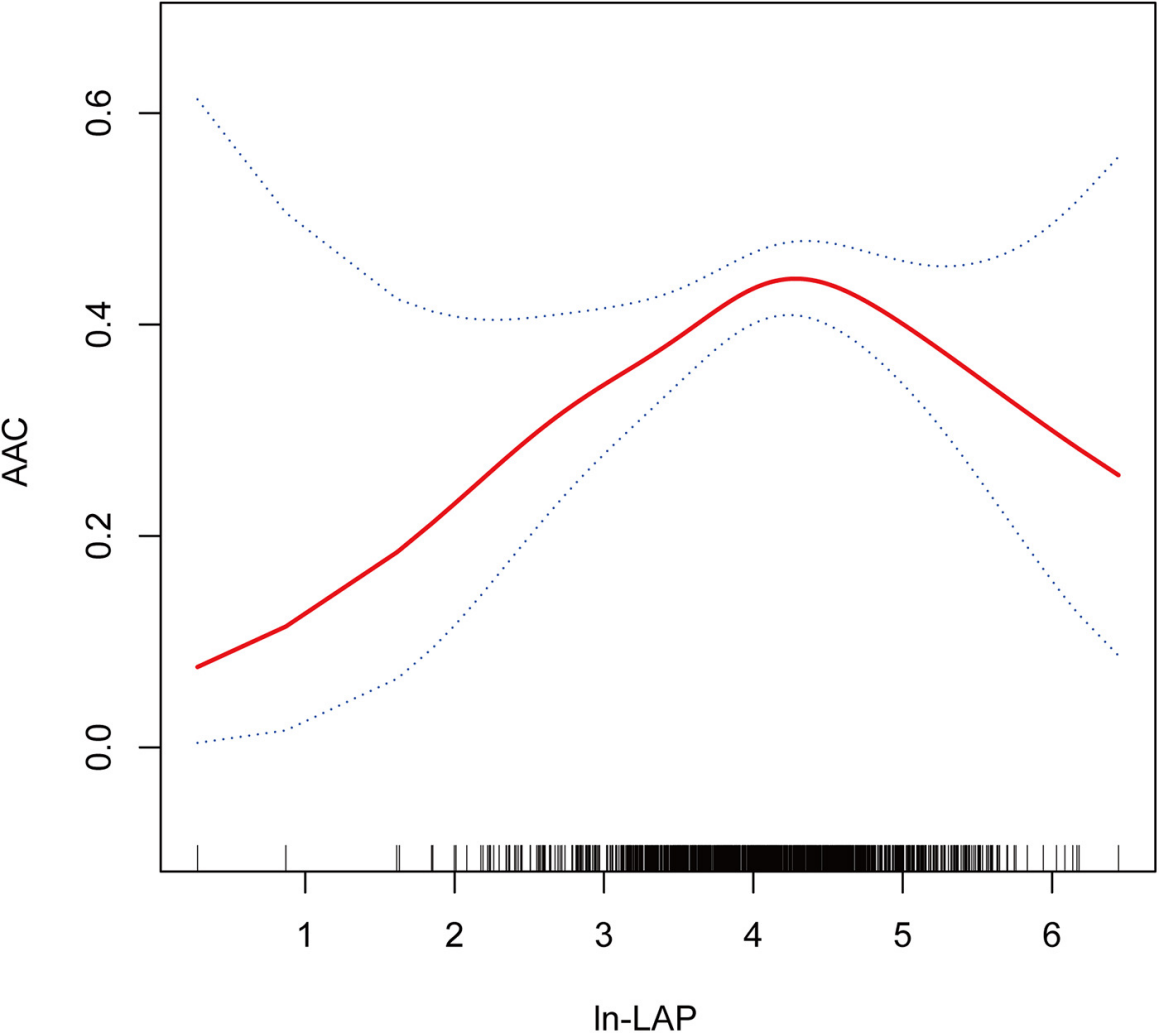
Dose-response relationship using a restricted cubic spline. Association between ln-LAP and AAC. The solid red line represents the estimated value and the blue dashed areas indicate their corresponding 95% confidence interval. All models were adjusted for age, gender, race, education level, FPL, marital status, BMI, hypertension, diabetes, smoking, alcohol consumption, hyperuricemia, and SB. ln-LAP, ln-transformed lipid accumulation product; AAC, abdominal aortic calcification; FPL, family poverty level; BMI, body mass index; SB, sedentary behavior.

Restricted cubic spline analyses (RCS) analysis demonstrates a nonlinear relationship between ln-LAP and AAC, characterized by an inverted U-shaped curve, as illustrated in Figure 2. As detailed in Table 4, threshold effect analysis identified a critical turning point at 4.21, which corresponds to an approximate LAP value of 67.8. Before this critical point, a robust positive correlation exists between ln-LAP and AAC (OR = 1.74; 95% CI: 1.21, 2.51; *P* < 0.01); however, beyond this point, the correlation shifts to a pronounced negative relationship (OR = 0.60; 95% CI: 0.39, 0.94; *P* < 0.05).

**Table 4:** Threshold effect analysis of the relationship between ln-LAP and AAC.

Outcome	AAC	*P*-value
	OR (95% CI)	
Model I		
One line effect	1.11 (0.89, 1.39)	0.37
Model II		
Inflection point (K)	4.21	
<K	1.74 (1.21, 2.51)	<0.01
>K	0.60 (0.39, 0.94)	<0.05
*P* for log-likelihood ratio test		0.001

Adjusted for age, gender, race, education level, FPL, marital status, BMI, hypertension, diabetes, smoking, alcohol consumption, hyperuricemia, SB. ln-LAP, ln-transformed lipid accumulation product; AAC, abdominal aortic calcification; FPL, family poverty level; BMI, body mass index; SB, sedentary behavior.

### 3.5 Sensitivity analysis

After excluding participants with extreme ln-LAP values (<2 or ≥6), the associations between higher ln-LAP and AAC/SAAC remained generally consistent (Supplementary Table S1). In the fully adjusted model, ln-LAP Q3 was significantly associated with increased odds of AAC (OR = 1.82; 95% CI: 1.14, 2.91; *P* = 0.012), and Q4 was associated with higher odds of SAAC (OR = 2.15; 95% CI: 1.07, 4.32; *P* = 0.031).

## 4 Discussion

In this study, we utilized the 2013–2014 NHANES dataset to assess the relationship between LAP and AAC. After adjusting for multiple covariates, ln-LAP was stratified into quartiles. The analysis demonstrated a significant correlation between Q3 and AAC compared to Q1 (OR = 1.91; 95% CI: 1.20, 3.04, *P* < 0.01), and a notable correlation with SAAC in Q4 (OR = 2.17; 95% CI: 1.08, 4.35, *P* < 0.05). Subgroup analyses, including factors such as age, gender, hypertension, and diabetes, indicated consistent correlations between ln-LAP and both AAC and SAAC, with no significant interactions observed (*P* for interaction >0.05). RCS analysis revealed that the relationship between ln-LAP and AAC is nonlinear, characterized by an inverse U-shaped curve. Threshold effect analysis identified 4.21 as the critical inflection point. Before this inflection point, ln-LAP and AAC exhibited a strong positive correlation (OR = 1.74; 95% CI: 1.21, 2.51; *P* < 0.01). Beyond this point, the correlation distinctly shifted to a negative relationship (OR = 0.60; 95% CI: 0.39, 0.94; *P* < 0.05).

AAC is common in the general population, with both its incidence and severity increasing with age. Studies have demonstrated that AAC serves as a more robust independent predictor of both all-cause mortality and cardiovascular disease than the Framingham risk score and coronary artery calcification (CAC) (4, 10). Epidemiologic studies and meta-analyses have substantiated that AAC is significantly correlated with an elevated risk of cardiovascular diseases, myocardial infarction, and stroke (3, 11–14). These findings underscore the critical importance of AAC in assessing cardiovascular risk.

Obesity, particularly visceral adiposity, plays a critical role in the development of cardiometabolic diseases and has been increasingly implicated in vascular calcification (15, 16). However, traditional obesity metrics such as BMI have significant limitations. BMI does not differentiate between fat and lean mass and poorly reflects central fat distribution. Indeed, previous studies found that BMI was not significantly associated with AAC after adjusting for confounders (17). Other anthropometric indices like waist-to-hip ratio (WHR) and waist-to-height ratio (WHtR) have shown stronger associations with AAC, with some evidence suggesting a nonlinear relationship (18). These findings underscore the importance of selecting appropriate markers of central obesity when evaluating vascular calcification risk.

In this context, LAP—a composite index derived from waist circumference and triglyceride levels—has emerged as a promising surrogate for visceral fat. Compared to BMI, WC, VAI, or WHtR, LAP offers several advantages: it reflects both anatomical and metabolic components of obesity, is inexpensive, easy to compute, and has been shown to predict cardiometabolic diseases such as diabetes, metabolic syndrome, and cardiovascular events more accurately (5, 6, 19–23). However, prior to this study, the relationship between LAP and AAC had not been systematically evaluated. Our findings indicate that, following the logarithmic transformation of LAP, significant associations were observed between AAC and the third quartile of ln-LAP, and similarly, between SAAC and the fourth quartile. Subgroup analyses confirmed that these correlations were not influenced by sex, age, hypertension, or diabetes. Threshold effect analysis identified K = 4.21 as the critical inflection point between ln-LAP and AAC, suggesting this point represents the peak risk for AAC. By demonstrating a novel and independent association between LAP and AAC, our study provides new insight into the metabolic determinants of vascular calcification and suggests that LAP may serve as a valuable tool for early identification of individuals at high cardiovascular risk.

The precise mechanisms underlying the correlation between the LAP and the risk of AAC remain unclear, yet several plausible explanations exist. Initially, lipid metabolism emerges as a key factor. This study confirmed that as a precise indicator of lipid accumulation and visceral obesity, LAP significantly correlates with AAC. Likewise, TG-related indicators, such as the triglyceride glucose index (TYG), also demonstrated correlations with AAC, further underscoring the crucial role of lipid metabolism in AAC formation. Additionally, lipid accumulation in the aortic wall is known to promote osteogenic differentiation and calcification of vascular smooth muscle cells (24). Animal studies involving LDL receptor-deficient mice have demonstrated that a high-fat diet activates bone morphogenetic protein 2 and escalates the production of reactive oxygen species, thereby inducing vascular calcification (25). Collectively, these studies highlight abnormalities in lipid metabolism and elucidate potential mechanisms through which LAP predicts AAC risk. An enhanced understanding of these mechanisms could offer novel perspectives and approaches for the prevention and management of AAC.

Secondly, LAP is a composite indicator linked to WC, with elevations typically indicative of obesity. Research has consistently demonstrated that various obesity indicators are significantly correlated with the progression of CAC (26–28). These findings further underscore the strong association between obesity and an elevated risk of AAC (29, 30). Typically elevated in obesity, leptin levels have been linked by a study from Pawel et al. to the severity and rapid progression of AAC (31). Experimental research has revealed that leptin induces both aortic calcification and the osteogenic differentiation of vascular smooth muscle cells (32, 33). The arterial wall is a potential target of leptin, which may promote the calcification process by influencing aortic mesangial vascular cells through specific receptors (34). Specifically, leptin enhances osteoblast-specific protein expression and calcification by activating the transcription factor Runx2 and inducing phosphorylation of kinases Erk-1 and Erk-2 (32, 34). Moreover, leptin amplifies these effects by boosting the expression of RANKL and bone morphogenetic protein 4 (35). Consequently, the synergistic interactions between lipid metabolism disorders and leptin may collectively contribute to the development and progression of AAC in individuals with higher LAP levels.

Finally, chronic inflammation and oxidative stress are key mechanisms influencing the formation of AAC (36, 37). Serving as an indicator of TG-associated lipid complexes, excessive TG levels in the LAP can activate immune cells, such as macrophages, through oxidation to form oxidized lipoproteins. Following the phagocytosis of oxidized lipoproteins, these immune cells transform into foam cells, releasing inflammatory factors and pro-inflammatory cytokines that exacerbate the inflammatory response of the vascular lining (38). Furthermore, oxidative stress escalates the production of free radicals, which may damage vascular cells and intensify inflammation and atherosclerosis (39). Elevated levels of the LAP may accelerate both the onset and progression of AAC by triggering chronic inflammatory and oxidative stress responses. Interestingly, the negative association observed beyond the inflection point (ln-LAP = 4.21) may be attributed to a combination of factors. These may include a limited sample size at extreme LAP levels, possible physiological adaptations that suppress calcification under severe metabolic stress, and the complex, nonlinear effects of lipid metabolism and inflammation on vascular calcification. The exact mechanisms underlying this association require further investigation.

This study is subject to the following limitations. First, the cross-sectional design of this study precludes the determination of a causal relationship between the LAP and AAC. Second, despite adjustments for multiple confounders, residual confounding factors may remain, potentially affecting the interpretation of the results. Additionally, the inability to assess whether modifications in LAP (e.g., through diet or treatment) would lead to corresponding changes in AAC is a limitation. Moreover, due to limitations of the NHANES database, a large number of participants were excluded because of missing AAC data, primarily related to DXA scan eligibility criteria. This may introduce selection bias and limit the robustness of subgroup analyses, particularly in smaller subpopulations such as individuals with diabetes. The reduced sample size in these subgroups may lead to wide confidence intervals and insufficient statistical power, thereby affecting the reliability and interpretability of some results. Finally, given the significant variation in AAC across different races (40, 41) and the study's limitation to US residents, the generalizability of the findings is potentially restricted. Therefore, to validate the reliability of these findings, larger international multicenter prospective studies are required.

## 5 Conclusions

This study identified a significant correlation between the LAP and AAC. Considering that AAC is a critical predictor of cardiovascular morbidity and mortality, early detection and intervention to delay its progression are imperative for reducing cardiovascular risk. As a reliable lipid composite, the LAP shows promise as a potential predictor of AAC. However, larger prospective studies are required to further substantiate LAP's clinical utility and validate its predictive value.

## Data Availability

The datasets presented in this study can be found in online repositories. The names of the repository/repositories and accession number(s) can be found below: https://www.cdc.gov/nchs/nhanes.

## References

[1] Bartstra JW Mali W Spiering W de Jong PA. Abdominal aortic calcification: from ancient friend to modern foe. Eur J Prev Cardiol (2021) 28(12):1386–91. 10.1177/2047487320919895 34647579

[2] Chuang ML Massaro JM Levitzky YS Fox CS Manders ES Hoffmann U Prevalence and distribution of abdominal aortic calcium by gender and age group in a community-based cohort (from the Framingham heart study). Am J Cardiol (2012) 110(6):891–6. 10.1016/j.amjcard.2012.05.020 22727181 PMC3432173

[3] Bastos Goncalves F Voute MT Hoeks SE Chonchol MB Boersma EE Stolker RJ Calcification of the abdominal aorta as an independent predictor of cardiovascular events: a meta-analysis. Heart (2012) 98(13):988–94. 10.1136/heartjnl-2011-301464 22668866

[4] Criqui MH Denenberg JO McClelland RL Allison MA Ix JH Guerci A Abdominal aortic calcium, coronary artery calcium, and cardiovascular morbidity and mortality in the multi-ethnic study of atherosclerosis. Arterioscler Thromb Vasc Biol (2014) 34(7):1574–9. 10.1161/ATVBAHA.114.303268 24812323 PMC4153597

[5] Kahn HS. The “lipid accumulation product” performs better than the body mass index for recognizing cardiovascular risk: a population-based comparison. BMC Cardiovasc Disord (2005) 5:26. 10.1186/1471-2261-5-26 16150143 PMC1236917

[6] Li Y Zheng R Li S Cai R Ni F Zheng H Association between four anthropometric indexes and metabolic syndrome in US adults. Front Endocrinol (Lausanne) (2022) 13:889785. 10.3389/fendo.2022.889785 35685216 PMC9171391

[7] Wang F Zheng J. Association between serum alpha-klotho and severe abdominal aortic calcification among civilians in the United States. Nutr Metab Cardiovasc Dis (2022) 32(6):1485–92. 10.1016/j.numecd.2022.02.017 35304049

[8] Zhou L Wen X Peng Y Guo M Zhao L. Red blood cell folate and severe abdominal aortic calcification: results from the Nhanes 2013–2014. Nutr Metab Cardiovasc Dis (2021) 31(1):186–92. 10.1016/j.numecd.2020.08.020 32988723

[9] Yu X Pu X Xi Y Li X Li H Zheng D. Association between the lipid accumulation product and chronic kidney disease among adults in the United States. Sci Rep (2024) 14(1):21423. 10.1038/s41598-024-71894-2 39271739 PMC11399144

[10] O'Connor SD Graffy PM Zea R Pickhardt PJ. Does nonenhanced Ct-based quantification of abdominal aortic calcification outperform the Framingham risk score in predicting cardiovascular events in asymptomatic adults? Radiology (2019) 290(1):108–15. 10.1148/radiol.2018180562 30277443

[11] Hendriks EJE de Jong PA Beulens JWJ van der Schouw YT Forbang NI Wright CM Annularity of aorto-iliac arterial calcification and risk of all-cause and cardiovascular mortality. JACC Cardiovasc Imaging (2018) 11(11):1718–9. 10.1016/j.jcmg.2018.01.029 29680342

[12] Chen HC Wang WT Hsi CN Chou CY Lin HJ Huang CC Abdominal aortic calcification score can predict future coronary artery disease in hemodialysis patients: a 5-year prospective cohort study. BMC Nephrol (2018) 19(1):313. 10.1186/s12882-018-1124-x 30409161 PMC6225627

[13] van der Meer IM Bots ML Hofman A del Sol AI van der Kuip DA Witteman JC. Predictive value of noninvasive measures of atherosclerosis for incident myocardial infarction: the rotterdam study. Circulation (2004) 109(9):1089–94. 10.1161/01.CIR.0000120708.59903.1B 14993130

[14] Levitzky YS Cupples LA Murabito JM Kannel WB Kiel DP Wilson PW Prediction of intermittent claudication, ischemic stroke, and other cardiovascular disease by detection of abdominal aortic calcific deposits by plain lumbar radiographs. Am J Cardiol (2008) 101(3):326–31. 10.1016/j.amjcard.2007.08.032 18237594

[15] Rhee EJ. The influence of obesity and metabolic health on vascular health. Endocrinology and Metabolism (2022) 37(1):1–8. 10.3803/EnM.2022.101 35255597 PMC8901957

[16] Wang JJ Zheng Z Zhang Y. Association of overweight/obesity and overweight/obesity-related metabolic dysfunction-associated steatotic liver disease in young adults with coronary artery calcification later in life. Diabetes Obes Metab (2024) 26(9):3860–7. 10.1111/dom.15733 38934214

[17] Fox CS Hwang SJ Massaro JM Lieb K Vasan RS O'Donnell CJ Relation of subcutaneous and visceral adipose tissue to coronary and abdominal aortic calcium (from the Framingham heart study). Am J Cardiol (2009) 104(4):543–7. 10.1016/j.amjcard.2009.04.019 19660609 PMC2723724

[18] Sun L. Associations between waist-to-height ratio and abdominal aortic calcification: a cross-sectional study. Medicine (Baltimore) (2024) 103(24):e38608. 10.1097/MD.0000000000038608 38875360 PMC11175898

[19] Ebrahimi M Seyedi SA Nabipoorashrafi SA Rabizadeh S Sarzaeim M Yadegar A Lipid accumulation product (Lap) index for the diagnosis of nonalcoholic fatty liver disease (Nafld): a systematic review and meta-analysis. Lipids Health Dis (2023) 22(1):41. 10.1186/s12944-023-01802-6 36922815 PMC10015691

[20] Yan P Xu Y Miao Y Tang Q Wu Y Bai X Association of lipid accumulation product with chronic kidney disease in Chinese community adults: a report from the reaction study. Lipids Health Dis (2021) 20(1):131. 10.1186/s12944-021-01569-8 34627270 PMC8502407

[21] Xia C Li R Zhang S Gong L Ren W Wang Z Lipid accumulation product is a powerful index for recognizing insulin resistance in non-diabetic individuals. Eur J Clin Nutr (2012) 66(9):1035–8. 10.1038/ejcn.2012.83 22781025

[22] Tian T Pei H Chen Z Hailili G Wang S Sun Y Comparison of lipid accumulation product and body mass index as indicators of diabetes diagnosis among 215,651 Chinese adults. PeerJ (2020) 8:e8483. 10.7717/peerj.8483 32095339 PMC7017788

[23] Raposo MA Guimaraes NS Tupinambas U. Lipid accumulation product index to predict metabolic syndrome in people living with Hiv. Clin Med Res (2020) 18(4):120–5. 10.3121/cmr.2020.1509 32340981 PMC7735448

[24] Demer LL Tintut Y. Inflammatory, metabolic, and genetic mechanisms of vascular calcification. Arterioscler Thromb Vasc Biol (2014) 34(4):715–23. 10.1161/ATVBAHA.113.302070 24665125 PMC3975044

[25] Derwall M Malhotra R Lai CS Beppu Y Aikawa E Seehra JS Inhibition of bone morphogenetic protein signaling reduces vascular calcification and atherosclerosis. Arterioscler Thromb Vasc Biol (2012) 32(3):613–22. 10.1161/ATVBAHA.111.242594 22223731 PMC3679546

[26] Kowall B Lehmann N Mahabadi AA Moebus S Erbel R Jockel KH Associations of metabolically healthy obesity with prevalence and progression of coronary artery calcification: results from the Heinz Nixdorf recall cohort study. Nutr Metab Cardiovasc Dis (2019) 29(3):228–35. 10.1016/j.numecd.2018.11.002 30648599

[27] Bacha F Edmundowicz D Sutton-Tyrell K Lee S Tfayli H Arslanian SA. Coronary artery calcification in obese youth: what are the phenotypic and metabolic determinants? Diabetes Care (2014) 37(9):2632–9. 10.2337/dc14-0193 25147256 PMC4392940

[28] Cassidy AE Bielak LF Zhou Y Sheedy PF 2nd Turner ST Breen JF Progression of subclinical coronary atherosclerosis: does obesity make a difference? Circulation (2005) 111(15):1877–82. 10.1161/01.CIR.0000161820.40494.5D 15837939

[29] Qin Z Du D Li Y Chang K Yang Q Zhang Z The association between weight-adjusted-waist index and abdominal aortic calcification in adults aged >/= 40 years: results from Nhanes 2013–2014. Sci Rep (2022) 12(1):20354. 10.1038/s41598-022-24756-8 36437292 PMC9701694

[30] Li W Wang Z Li M Xie J Gong J Liu N. Association between a body shape index and abdominal aortic calcification in general population: a cross-sectional study. Front Cardiovasc Med (2023) 9:1091390. 10.3389/fcvm.2022.1091390 36704474 PMC9871763

[31] Szulc P Amri EZ Varennes A Panaia-Ferrari P Fontas E Goudable J Positive association of high leptin level and abdominal aortic calcification in men- the prospective minos study. Circ J (2018) 82(12):2954–61. 10.1253/circj.CJ-18-0517 30282882

[32] Zeadin M Butcher M Werstuck G Khan M Yee CK Shaughnessy SG. Effect of leptin on vascular calcification in apolipoprotein E-deficient mice. Arterioscler Thromb Vasc Biol (2009) 29(12):2069–75. 10.1161/ATVBAHA.109.195255 19797706

[33] Rosa M Paris C Sottejeau Y Corseaux D Robin E Tagzirt M Leptin induces osteoblast differentiation of human valvular interstitial cells via the Akt and Erk pathways. Acta Diabetol (2017) 54(6):551–60. 10.1007/s00592-017-0980-3 28314924

[34] Parhami F Tintut Y Ballard A Fogelman AM Demer LL. Leptin enhances the calcification of vascular cells: artery wall as a target of leptin. Circ Res (2001) 88(9):954–60. 10.1161/hh0901.090975 11349006

[35] Liu GY Liang QH Cui RR Liu Y Wu SS Shan PF Leptin promotes the osteoblastic differentiation of vascular smooth muscle cells from female mice by increasing Rankl expression. Endocrinology (2014) 155(2):558–67. 10.1210/en.2013-1298 24248461

[36] McEvoy JW Nasir K DeFilippis AP Lima JA Bluemke DA Hundley WG Relationship of cigarette smoking with inflammation and subclinical vascular disease: the multi-ethnic study of atherosclerosis. Arterioscler Thromb Vasc Biol (2015) 35(4):1002–10. 10.1161/ATVBAHA.114.304960 25745060 PMC4484586

[37] Al Hariri M Zibara K Farhat W Hashem Y Soudani N Al Ibrahim F Cigarette smoking-induced cardiac hypertrophy, vascular inflammation and injury are attenuated by antioxidant supplementation in an animal model. Front Pharmacol (2016) 7:397. 10.3389/fphar.2016.00397 27881962 PMC5101594

[38] Hou P Fang J Liu Z Shi Y Agostini M Bernassola F Macrophage polarization and metabolism in atherosclerosis. Cell Death Dis (2023) 14(10):691. 10.1038/s41419-023-06206-z 37863894 PMC10589261

[39] Sena CM Pereira A Fernandes R Letra L Seica RM. Adiponectin improves endothelial function in mesenteric arteries of rats fed a high-fat diet: role of perivascular adipose tissue. Br J Pharmacol (2017) 174(20):3514–26. 10.1111/bph.13756 28236429 PMC5610162

[40] Allison MA Budoff MJ Nasir K Wong ND Detrano R Kronmal R Ethnic-specific risks for atherosclerotic calcification of the thoracic and abdominal aorta (from the multi-ethnic study of atherosclerosis). Am J Cardiol (2009) 104(6):812–7. 10.1016/j.amjcard.2009.05.004 19733716 PMC2755558

[41] Forbang NI McClelland RL Remigio-Baker RA Allison MA Sandfort V Michos ED Associations of cardiovascular disease risk factors with abdominal aortic calcium volume and density: the multi-ethnic study of atherosclerosis (mesa). Atherosclerosis (2016) 255:54–8. 10.1016/j.atherosclerosis.2016.10.036 27816809 PMC5801758

