# Supplementary material for: Lipid accumulation product index is inversely U-shaped associated with abdominal aortic calcification based on NHANES 2013–2014
Source: Front Cardiovasc Med. 2025 Jun 9;12:1524847. doi: 10.3389/fcvm.2025.1524847 (PMC12183298; doi:10.3389/fcvm.2025.1524847)
Supplement: Supplementary file 1 [file Supplementaryfile1.docx]

Supplementary Material

# Supplementary Figures and Tables

Table S1. Sensitivity analysis of the association between ln-LAP and AAC score, AAC, and SAAC (excluding participants with ln-LAP < 2 or ≥ 6)

|  | Model 1 β/OR (95% CI) | *P*-value | Model 2 β/OR (95% CI) | *P*-value | Model 3 β/OR (95% CI) | *P*-value |
| --- | --- | --- | --- | --- | --- | --- |
| AAC score |  |  |  |  |  |  |
| ln-transformed LAP (categories) |  |  |  |  |  |  |
| Q1 | 0 |  | 0 |  | 0 |  |
| Q2 | 0.38 (0.02, 0.74) | 0.04 | 0.13 (-0.20, 0.47) | 0.436 | 0.15 (-0.63, 0.92) | 0.708 |
| Q3 | 0.42 (0.05, 0.78) | 0.025 | 0.16 (-0.18, 0.50) | 0.348 | 0.33 (-0.48, 1.15) | 0.424 |
| Q4 | 0.39 (0.02, 0.75) | 0.038 | 0.15 (-0.19, 0.49) | 0.374 | 0.40 (-0.48, 1.27) | 0.375 |
| AAC |  |  |  |  |  |  |
| ln-transformed LAP (categories) |  |  |  |  |  |  |
| Q1 | 1 |  | 1 |  | 1 |  |
| Q2 | 1.40 (1.11, 1.76) | 0.004 | 1.28 (1.00, 1.63) | 0.054 | 1.13 (0.72, 1.76) | 0.596 |
| Q3 | 1.51 (1.20, 1.89) | 0.001 | 1.39 (1.08, 1.78) | 0.01 | 1.82 (1.14, 2.91) | 0.012 |
| Q4 | 1.36 (1.08, 1.71) | 0.01 | 1.25 (0.97, 1.60) | 0.084 | 1.32 (0.80, 2.17) | 0.282 |
| SAAC |  |  |  |  |  |  |
| ln-transformed LAP (categories) |  |  |  |  |  |  |
| Q1 | 1 |  | 1 |  | 1 |  |
| Q2 | 1.35 (0.92, 1.97) | 0.126 | 1.17 (0.77, 1.76) | 0.462 | 1.36 (0.73, 2.52) | 0.329 |
| Q3 | 1.41 (0.97, 2.05) | 0.074 | 1.24 (0.82, 1.87) | 0.305 | 1.58 (0.83, 3.01) | 0.16 |
| Q4 | 1.46 (1.00, 2.13) | 0.048 | 1.41 (0.94, 2.13) | 0.101 | 2.15 (1.07, 4.32) | 0.031 |

Model 1 was adjusted for none. Model 2 was adjusted for age, gender, and race. Model 3 was adjusted for age, gender, race, education level, FPL, marital status, BMI, hypertension, diabetes, smoking, alcohol consumption, hyperuricemia, and SB. Abbreviations: Q1, Quartile 1; Q2, Quartile 2; Q3, Quartile 3; Q4, Quartile 4; ln-LAP, ln-transformed lipid accumulation product; AAC, abdominal aortic calcification; SAAC, severe abdominal aortic calcification; FPL, family poverty level; BMI, body mass index; SB, sedentary behavior.
